# Energy Management Education in Persons with Long COVID-Related Fatigue: Insights from Focus Group Results on Occupational Therapy Approach

**DOI:** 10.3390/healthcare12020150

**Published:** 2024-01-09

**Authors:** Andrea Weise, Eliane Ott, Ruth Hersche

**Affiliations:** 1 Rehabilitation Research Laboratory 2rLab, Department of Business Economics, Health and Social Care, University of Applied Sciences and Arts of Southern Switzerland, 6928 Manno, Switzerland; 2 Rehabilitation Center Basel (REHAB), 4055 Basel, Switzerland

**Keywords:** fatigue, long COVID-19, occupational therapy, self-management, rehabilitation

## Abstract

Background: Long COVID is a growing condition among individuals, with fatigue being one of the main symptoms experienced. Energy Management Education (EME) is a structured occupational therapy group intervention that aims to reduce the impact of fatigue in daily life. Methods: This study utilized focus groups to explore the experiences of individuals with post-COVID-related fatigue who participated in the EME program. Six participants engaged in discussions about the program immediately after its completion and again two months later. Additionally, five occupational therapists shared their experiences. Results: Former participants reported implementing strategies learned in the program to manage their condition effectively. They emphasized the importance of understanding fatigue and found the support provided by the peer group valuable. Occupational therapists highlighted the unique challenges faced by individuals with post-COVID-related fatigue compared to other populations with similar fatigue symptoms. Furthermore, insights were obtained regarding the ways individuals live and cope with post-COVID-related fatigue. Conclusion: EME participants were involved in a dynamic and complex occupational therapy process and were experiencing a slow change towards having more control over their daily routines. The study gathered valuable feedback and suggestions from participants and occupational therapists which can be used to optimize the EME program.

## 1. Introduction

The coronavirus disease 2019 (COVID-19) is an infection causing an acute respiratory syndrome. Symptoms persisting after COVID-19, lasting longer than 12 weeks, that cannot be explained by another diagnosis are defined as a post-COVID-19 syndrome (PCS) [1] and are commonly referred to as long COVID [2]. Current long COVID prevalence estimates range from 7.5% to 41% in non-hospitalized adults, 2.3–53% in mixed adult samples, and 37.6% in hospitalized adults [3]. The symptoms of PCS may be singular, multiple, constant, transient, or fluctuating and can change in nature over time [4]. The most common symptoms are fatigue, post-exertional malaise, and breathlessness. Individuals with PCS often encounter profound fatigue, which can severely affect their abilities and hinder participation in complex or social daily activities [5]. This fatigue not only causes distress but also acts as a barrier, preventing a return to previous routines, work, and social engagements [6]. 

Until now, recommendations for managing PCS are primarily based on expert opinions and the lived experiences of patients. These recommendations stress the importance of a multidisciplinary approach, incorporating self-management education, peer support, and strategies for managing symptoms [1]. Occupational therapists (OTs) can draw from best practices and self-management education protocols for fatigue related to chronic diseases, such as multiple sclerosis, cancer survivorship, and rheumatoid arthritis. These interventions have demonstrated positive effects on fatigue impact, occupational performance, and quality of life in individuals with these conditions [5,6,7].

Energy Management Education (EME) [8] is an evidence-based [9,10] and occupational therapy-based group intervention developed originally for persons with multiple sclerosis-related fatigue in the Swiss healthcare system. As the energy management strategies are not disease specific, a disease-independent version was created after a comprehensive literature review of self-management education in different disease populations.

Since the beginning of the COVID-19 pandemic, OTs have functioned in treating people with acute and post-acute COVID-19 and PCS in different settings (acute care, inpatient rehabilitation, and outpatient reintegration). Some EME-trained OTs have started to include persons with PCS-related fatigue in their EME groups or used the EME materials as part of individual therapy. In early summer 2021, nine OTs from different clinical settings that treat persons with PCS-related fatigue shared their growing experiences using the EME in this new patient group. They reported that the EME protocol is feasible and appropriate; however, optimization for persons with long COVID-related fatigue is required [11]. In person-centered healthcare services, a crucial aspect is understanding and valuing the perspective and experiences of customers. Therefore, prior to commencing a review and an adaptation of the EME materials, it was essential to gather the experiences and feedback from former EME participants who are affected by PCS-related fatigue. 

The primary aim was to enhance the scientific basis and improve a self-management education program for those coping with PCS-related fatigue. Additionally, it sought to refine EME-training for occupational therapists, enabling evidence-based patient education in this field. Specifically, the study aimed to evaluate content suitability, pinpoint any potential gaps, and gather insights into the daily life changes experienced by former EME participants with PCS-related fatigue in Switzerland through focused group discussions.

## 2. Materials and Methods

Focus group research involves engaging a small number of people in an informal group discussion focused on a particular topic or set of issues. An expert moderator that stimulates an open atmosphere, perceptions, ideas, opinions, and thoughts leads the discussion [12]. All participants provided informed consent. All participants’ names and quotes are anonymized.

### 2.1. Setting

Since March 2021, the Rehabilitation Center Basel (REHAB) has provided an interprofessional consultation and an outpatient treatment program for persons with ongoing symptoms at least four weeks after COVID-19 infection. After registration, the symptoms are recorded. Based on the symptom cluster and the rehabilitation goals, the inter-professional team creates a 6–9-week treatment plan tailored to the individual patient. EME is one of the rehabilitation interventions, together with physical exercise training, mindfulness, and consultations by different health professionals (e.g., cardiologists, pulmonologists, psychologists, and social workers), aimed to accompany people with PCS on their way back to normality. EME aims to increase self-management skills in managing available energy and achieving a satisfying and meaningful daily routine despite fatigue. Participants learn about the factors influencing their energy level and experiment with skills to manage their energy, using behavioral strategies (e.g., pace; plan; prioritize activities; and optimize their communication, environment, and ergonomic behavior). Led by a trained OT, they identify and implement tailored behavior modifications and adapt their habits and routines.

The inclusion criteria for EME are being experienced in living with fatigue (FSS, score ≥ 4) [13], no major depression (BDI-FS, <8) [14], no major cognitive impairments (MOCA, ≥26) [15], and sufficient linguistic ability and motivation for self-management education in the German language. EME starts with a first individual session (45 min), followed by five group sessions (duration of 90 min, including a 15 min break; max 5 participants), a subsequent individual session (45 min), and then a booster mail eight weeks after the last session (Table 1). Five OTs from the rehabilitation center are trained in conducting EME and have experience in treating persons living with fatigue due to PCS and other underlying conditions.

### 2.2. Focus Group Participants

We planned to enroll and include nine EME participants with PCS-related fatigue. The inclusion criteria were to provide informed consent, have attended at least six out of seven sessions, and be willing to participate in a focus group discussion straight after the conclusion of EME and in an online focus group two months later. The online focus group addressed the same group of individuals. All five EME trained OTs from the rehabilitation center conducting EME sessions with persons with PCS-related fatigue were invited to participate in one focus group discussion. 

### 2.3. Focus Group Discussions

The focus group discussions after the completion of EME were scheduled twice in a quiet room of the rehabilitation center (*n* = 4, *n* = 5). The date of the online focus group was communicated considerably in advance; we assumed, however, that we would reach less of them due to health and personal commitments.

In line with the study’s objectives and the multifaceted nature of occupational therapy as a dynamic process [16], three distinct interview guides were created: one completing EME; one two months post-intervention, both with the same subjects; and one for EME occupational therapists. These guidelines were crafted with input from prior research findings [13], informal discussions with OT practitioners, and insights gleaned from the scientific literature focusing on the life experiences of individuals with PCS [17] (see Supplementary Materials).

Each focus group discussion followed a structured format, consisting of an introductory phase, a main discussion segment, and a concluding phase. The focus group after the seventh EME session involved participants sharing personal experiences, evaluating the relevance of EME themes and materials, and suggesting potential improvements. Two months later, these same participants offered insights into the practical application of EME strategies in their daily lives and recommended further enhancements. In the discussion with occupational therapists (OTs), the focus was on addressing the specific requirements of individuals with PCS during EME, effective modifications to the EME protocol, and brainstorming ideas for future improvements.

RH led the focus groups with EME participants, supported by AW as a co-moderator. Conversely, in the OT focus group, AW took the lead role, with RH as the co-moderator. Both RH and AW possess extensive experience in facilitating groups and are adept at using qualitative research methods to explore the perspectives of healthcare providers and users. Following each focus group, the co-moderator summarized the key discussion points (member check) and encouraged participants to ensure the completeness of the data or add any missed elements. Subsequently, immediate debriefings were conducted by the researchers. All discussions and debriefings were recorded for analysis purposes.

### 2.4. Analysis

All conversations were transcribed by a research assistant and cross-checked against field notes by AW. These transcripts formed the basis for analyzing the focus group discussions. AW conducted a thorough thematic analysis using a structured six-step process [18]. Initially, codes were created to capture essential concepts and then organized into categories. Prioritization was based on factors such as quote frequency, intensity, extensiveness, and specificity. These categories were then amalgamated into overarching themes, aligning them with the complex dynamic process model in occupational therapy [16]. This preliminary system was shared and discussed until a consensus was reached by AW and RH. 

## 3. Results

In January 2022, six EME participants participated in one of the two focus group discussions after session 7 (duration 60 min) (*n* = 2, *n* = 4), while three participants canceled their participation on short notice due to health issues. In March 2022, five out of the six focus group participants from January joined the online focus group (duration 50 min), while one person could not join due to Internet unavailability. All five EME OTs participated in the focus group discussion in January 2022 (duration of 60 min).

### 3.1. EME Group Participants and EME Occupational Therapists

The sample of the EME group participants is composed of five women and one man, with a mean age of 54.7 (SD: 11.6). On average, there were 11.2 months between COVID-19 infection and the EME start (Table 2). The sample of EME OTs is composed of five women with a mean age of 48 years (SD, 11.2; min–max, 32–58). Their professional experience varies between 7 and 34 years (mean, 22; SD, 11.8).

### 3.2. Focus Group Results: EME Group Participants

Three main themes emerged from the data with the EME-group participants. The experiences with EME and recommendations to health professionals are directly related to the intervention context. The third theme arises from the person-in-context and shows the tension with the broader macro context (see Table 3).

#### 3.2.1. Experiences with EME

Program: The EME program had a significant impact on the participants’ understanding of fatigue and their personal strategies for managing it. The structured nature, group dynamic, and workbook were highlighted as positive aspects. Participants appreciated the increased knowledge about fatigue, the reflection on their personal experiences, and the identification of effective strategies. Some participants saw tangible results, like fewer energy crashes and re-engagement in valued activities.

However, there were also some challenges identified. The workbook’s portability and the need for more tailored content for individuals dealing with long COVID were noted as areas for improvement. Additionally, the length of the group sessions was seen as too long for some participants. Some felt the focus on physical energy did not align with their specific experiences, and despite significant changes, a few individuals did not see substantial improvements.

The persistence of using acquired strategies after two months and the establishment of new daily routines were positive outcomes. Participants recognized the difficulty of applying these strategies in new or unexpected situations, like planning during holidays, highlighting the ongoing challenges of managing fatigue.

The group setting remained essential for success, emphasizing the support and accountability it provided. However, one participant mentioned that the strategies seemed to work only in times when fatigue was less pronounced, indicating a potential limitation in their effectiveness during more challenging periods.

*(…) the contact within the group and the exchange were essential.*

*(1st interview, January 2022: Mrs. Schneider)*

*I would be at another point without EME.*

*(2nd int., March 2022: Mrs. Keller)*

The participants had several insightful recommendations after completing the EME program. They suggested offering this group therapy early in the disease stage, ideally for smaller groups, and combining a workbook with a digital application for enhanced accessibility and engagement. Adding a session involving relatives and incorporating the cognitive aspect of daily activities were seen as valuable additions.

There was a consensus among participants that setting realistic and personalized goals at the end of each session could have aided them in implementing behavior changes effectively. After two months, the idea emerged to split the EME program: starting with an individual lesson focusing on fatigue symptoms and its impacts and then introducing group therapy once patients have more experience and acceptance.

Furthermore, preparing participants for a potentially long journey ahead and emphasizing the value of their lives beyond specific activities or roles, especially if disturbed due to illness, were recommended.

External factors like navigating insurance procedures and addressing social influences that participants have little control over should also be integrated into the program. Additionally, advice on coping with negative thoughts and stress was highlighted as essential for comprehensive support.

*One lesson including relatives would be helpful.*

*(1st int.: Mrs. Schneider)*

*A combination of an app with a workbook would have saved energy.*

*(1st int.: Mrs. Schmid)*

Occupational therapists: Participants were overall positive about the chairing of the EME groups, especially the high esteem felt towards everyone’s experiences and efforts. Some participants found that some therapists kept too close to the program at the expense of other communication unrelated to the topic of the specific therapy session. Whenever the group’s need for spontaneous communication around fatigue was met, the satisfaction of those group members rose. Two participants expressed the need for parallel psychological group therapy. Two months later, the same topics arose, and, additionally, the recommendation was expressed that the program should be a guideline but not followed too strictly. Space for their needs was expected.

*Some followed the chapters too strictly, but others took up the group’s needs more spontaneously.*

*(1st int.: Mr. Müller)*

Contents of sessions: The EME program left a lasting positive impact, particularly Lesson 1 on fatigue and Lesson 6 on communication, which were highlighted as essential even two months after completion.

In the focus group following the completion of the EME program, participants felt that Lesson 4 (body and environment) was less focused on their specific issues. Consequently, they recommended combining Lessons 4 and 5 into a single session and suggested moving Lesson 6 to an earlier stage of the program. Additionally, the example of a filled-in week plan provided in Lesson 3 was widely discussed as frustrating and challenging. It was perceived to represent their former life or an ideal activity level to strive for. As a suggestion, they proposed replacing it with one or two more realistic, less intense examples.

*The knowledge about fatigue in the first lesson helped me understand what is going on.*

*(1st int.: Mr. Müller)*

*The image of the bank account initiated a shift in my thinking.*

*(2nd int.: Mr. Förster)*

*The prepared week plan does not work for me because my energy is multidimensional, not linear.*

*(1st int.: Mrs. Schmid)*

Energy management strategies and tools: Participants found varying effectiveness in energy management strategies and tools post-EME. The weekly plan was deemed impractical due to unpredictable fatigue and low energy levels for some. Standardized sentence usage for returning questions was not universally effective, yet it saved energy for others. The metaphor of a bank account did not resonate with one participant due to its oversimplification, though it aided others in visualization and communication.

Several strategies proved beneficial: prioritizing tasks, spacing out activities, undertaking demanding tasks consecutively, and working at a manageable pace while managing breaks effectively led to fewer energy crashes. Including pleasurable activities boosted life satisfaction. Implementing energy-efficient body positions and tools, such as using aids while walking, helped conserve energy. Improved communication strategies fostered better support from immediate social circles, with some participants exploring task delegation. 

After two months, planning, prioritizing, breaks management, effective communication, energy-efficient positions, and tool usage remained helpful. A better understanding of fatigue and its influencing factors was seen as crucial. However, three participants found the example of the weekly plan in the workbook to be impractical or not applicable.

Self-training tasks: Participants initially found concrete and practical self-training tasks beneficial between therapy sessions. One participant specifically mentioned selecting tasks she anticipated would be most impactful for her. Additionally, there was a suggestion to allocate room for self-formulated tasks after each lesson. However, after two months, there was no further mention of the self-training tasks.

*I picked out those tasks from which I expected to benefit.*

*(1st int.: Mrs. Meier)*

#### 3.2.2. Recommendations for Health Professionals

Information: After completing EME, participants expressed a desire for earlier and more comprehensive information from healthcare professionals regarding long COVID’s impact on individuals. They also wished for professionals to inform their relatives rather than them having to take on that responsibility. They advocated for medication details, including for fatigue and depression, to be included in the basic information provided to those affected by long COVID.

However, after two months, the focus shifted. There was no further mention of informing the general population or relatives. Instead, participants emphasized the need for detailed information about medications, deeper insights into their symptoms, and timely responses to their queries from an early stage. They recommended informing affected individuals early on about the potential extended duration of their condition and offering insights into potential treatment options, like EME, if necessary.

*I wish that we would not have to do the education of our relatives and others about what is going on.*

*(1st int.: Mrs. Meier)*

Rehabilitation: Participants discussed missing parts in and timing of treatment options and about recognition of individual experiences and resources. They mentioned resilience; mindfulness; dealing with negative, self-destructive thoughts; and knowledge about a correct fitness training strategy with fatigue needing more attention in intervention programs. The opinion that persons with long COVID should be referred to specialized treatment much earlier was shared in all discussions. They recommended participation in EME groups at an earlier rather than a later stage, but not before gaining some acceptance of the disease and some experience with the symptoms in their personal life. Participants pointed out that, despite being part of standardized treatment programs, participants’ individual experiences and resources should be recognized and used.

*Right in the beginning, the referral to an EME group would be essential, but it is too early. However, it may be possible to give an outlook that this could be an option later.*

*(2nd int.: Mrs. Keller)*

#### 3.2.3. Living with Long COVID 

Sharing experiences about long COVID and expectations after the COVID-19 infection was a real need during the post-EME focus groups. Two months later, the need to explain was less urgent, and the discussion was more articulated around the specific questions about EME. 

In the discussions following EME, participants outlined the primary symptoms of long COVID and vividly described their profound impact. Everyday activities that were once normal became challenging or impossible, such as household chores; work; childcare duties; and simple leisure activities, like going to the cinema or a restaurant. They also mentioned experiencing lapses in memory or awareness, like missing bus stops, leading to a loss of self-assurance and control in their lives.

Two months later, symptoms like brain fog, cognitive limitations, fatigue, unpredictable physical restrictions, breathing issues, and stress continued to dominate their experiences. These symptoms still significantly restricted their daily lives, affecting their physical resilience, limiting their weekly plans, and fostering a sense of helplessness. Moreover, they expressed losing confidence in their abilities and a sense of achievement, finding it distressing to continuously explain their condition to friends and relatives who struggle to understand.

*We were catapulted out of a busy, active, resourceful everyday routine.*

*(1st int.: Mr. Förster)*

*Fatigue is my biggest problem. It wrestles me to the ground in all kinds of situations. It is hell.*

*(2nd int.: Mr. Müller)*

After finishing EME, participants described that their illness insight had been a long road and is still challenging, especially in ‘prime times’. Very slowly, they begin to improve and are somewhat able to count on improved phases each day. New daily routines were integrated into everyday life, like going to a psychologist weekly, checking their heartbeat regularly, taking breaks between two activities, and doing things slower. Two months after EME, they were still busy optimizing and adapting but focused more on details. They described accepting instead of fighting as their most important step in the past. The stages of mourning were mentioned. They discovered that their life is worth living now, not only when they are healthy again.

*Two months after EME, I am still optimizing, but it is just fine-tuning.*

*(2nd int.: Mrs. Keller)*

After COVID-19 infection, many of the participants had experienced being left alone and overloaded with well-meaning ideas about what would be the right way for them to deal with the situation. They had expected a quicker recovery. They reported that this very slow recovery process with long stabilizing phases without any progress is hard to bear. Earlier professional supervision had helped to accept this slow process without guilt. They wished for earlier contact with specialized health professionals who had accompanied their recovery, e.g., the reintegration into work. Two months later, participants better understand their condition and possible triggers, e.g., that unforeseen situations trigger stress, and stress triggers respiration problems, fatigue, or crashes.

*(…) overloaded with 1000 opinions of others, what would do me good, e.g., kinesiology, laying down on the ground in a forest, and so on. That made me crazy.*

*(1st int.: Mr. Müller)*

*I wanted to progress, but I was caught in this very slow step-by-step process. That needed much patience and was hard to bear and manage.*

*(1st int.: Mrs. Keller)*

### 3.3. Focus Group Results: EME Occupational Therapists 

The data that emerged from the focus group with the OTs are divided into three sub-themes: experiences with people with PCS in EME group therapy, reflections on EME occupational therapists’ role, and improvements of the EME program and materials. These themes belong to the therapist-in-context, which centers on factors like the institutional environment and professional competence and roles [16] (p. 14).

Compared to other clients in EME groups, e.g., those with multiple sclerosis, OTs reported that people with PCS are less experienced with fatigue in their everyday lives. Their daily energy is hardly predictable for them and comes with unpredictable daily changes. Most inspiring activities have disappeared or are substituted by other, more straightforward activities to keep busy or distracted. Some participants are cognitively and/or emotionally severely challenged by patient education. Overall, they need a lot more time to arrive in the group, describe emotional states, take up information, and implement new insights. 

The OTs pointed out that participating in EME sometimes takes effect only months later. The most important aspect during EME was described as ‘gaining awareness’: what fatigue means, that it is invisible to others, how their fatigue impacts their everyday life, and what a break is. The topics of breaks and communication were the two most important. Within the group setting, people with PCS feel understood and profit from each other’s experience. A key factor for success within EME with these clients’ groups is the exchange between each other.

*With other patients with fatigue, you can build up on a somehow stable situation. That is not possible with these clients. When they get up in the morning, they never know how much energy they will have for that day.*

*(Nadine)*

The OTs reflected on their EME group moderation with people with PCS. They found it more challenging than with other client groups. In their eyes, three aspects are mainly responsible: participants need more time for most aspects of group therapy; participants are less experienced with fatigue, which is also somewhat unpredictable; and dealing with emotions is more time-consuming. OTs must deal adequately with budding emotions and are convinced that a demarcation to psychotherapy is essential. Therefore, smaller numbers of participants and steady groups were recommended. They mentioned their need for super- or inter-vision regularly while leading EME groups with people with PCS. Additionally, they wished for more knowledge and skills to be imparted during EME education about group moderation, dealing with participants’ emotions in a group setting, and handling the strain as a group therapist.

*Five participants in the group sometimes are too many because everything needs more time.*

*(Stefanie)*

*(…) e.g., how to deal professionally with group members that take up much space in the group.*

*(Sabine)*

The participating therapists discussed a wide range of changes and optimizations in the structure and contents of EME for groups with people with PCS. They would like some themes to be joined, some to be expanded or newly introduced, and for some to be introduced at a different point in the program. Also, recommendations on specific details were given.

*The topic ‘breaks’ should be the first group session for every participant, and ‘communication’ should appear earlier in the program.*

*(Stefanie)*

*Exemplary breaks like breathing or awareness exercises, short meditations, or getting some air should be part of every session.*

*(Julia)*

*The planning strategy should be handled in another way due to the unpredictability of these patients’ energy. (…)*

*(Annika)*

The recommended improvements can be organized into four topics: slimming down, expanding, layout, and media. The EME therapists wished for less information in the workbook for EME participants, especially within the chapter for session one. They also recommended a different approach to self-training: instead of fixed tasks per session, two empty spaces—one for a self-formulated take-home message and one for a self-formulated goal or intention until the next session. They recommended additional information or a new session for relatives about emotions, cognitive fatigue, and related strategies, as well as pointers on relevant apps and websites. Concrete tips for optimizing the visualization and layout included examples for filled-in plans with lesser activities and more breaks. They also recommended extending the materials with apps for specific tasks and contents.

*The enclosed example of a weekly plan is too plain and should be replaced with a slimmer one. The enclosed costs too much time and patients get frustrated compared to their life situation.*

*(Stefanie)*

*One lesson for relatives or a summary for them (…) would save patients much energy.*

*(Sabine)*

## 4. Discussion

This study explored the experiences of individuals dealing with PCS-related fatigue within the EME program, capturing their post-program recommendations after a two-month follow-up. It also leveraged the expertise of seasoned EME therapists to enhance the intervention’s protocol, materials, and implementation within an institution. 

The findings of this study revealed the dynamic and multifaceted occupational therapy process experienced by EME participants, highlighting their engagement and gradual progress towards regaining control over daily habits and routines. In the context of intervention, the EME program showed how the interpersonal and dynamic interactions between participants and therapist fostered esteem and respect among participants, and the focus on daily routines increased knowledge about fatigue and the practical exploration of daily activity strategies. However, areas for improvement were also identified, including the need to address cognitive fatigue, introduce sessions for relatives, provide a more comprehensive explanation of post-exertional malaise, and tailor workbook examples to better suit participant needs. 

Further, the findings showed how the macro context, like the environment, the media, the government, or global events, has an ongoing influence on person, therapist, and intervention contexts. In fact, individuals with PCS voiced concerns about insufficient information from knowledgeable healthcare professionals and the absence of clear treatment pathways. This emphasizes the connection between the study’s findings and the broader context of the COVID-19 pandemic, highlighting the difficulties of the Swiss healthcare system to address the specific needs of individuals with PCS in a timely manner. The desire for earlier information on PCS-related fatigue stemmed from the fact that, on average, the COVID-19 infection occurred about a year before the start of the interdisciplinary rehabilitation program. The healthcare system’s management of their condition lacked coordination over months, and communication often lacked coherence and clarity, aligning with findings from a recent online survey from Switzerland [17]. These care trajectories correspond to the experiences outlined by Wurz et al. [19] and McNabb [20], pinpointing two domains of unmet needs: healthcare and social and emotional support. These experiences are instrumental in shaping the long-COVID phenomenon, marking it as the inaugural patient-driven illness. In this context, thousands of patients collectively shed light on a range of heterogeneous and intricate symptoms, many of which were not widely recognized within various healthcare and policy channels during the initial stages of the pandemic [21].

Recommendations to health professionals to be more and better informed and aware of the specifics of people with PCS underline the importance of their expertise of them in general, especially of OTs in the case of interventions in PCS-related fatigue. Ideally, EME should start sufficiently early to avoid the feeling of being let alone and exposed to uncertainty. However, persons must have experienced fatigue over a certain time, the burden of the consequences must have impacted their daily life, and they must have started to accept that the prognosis regarding recovery and healing might be uncertain. In these conditions, an intensive self-management education like EME in an outpatient clinic can empower participants to think about and engage in behavior changes. In an earlier stage, people who cannot sustain the exertion of a group session or those with fatigue experience for only a few weeks should receive basic information on fatigue and break management during the first individual occupational therapy sessions at the primary care level [22]. Furthermore, the option of a group intervention should be presented, if needed later. It needs a coordinated and integrated approach that shows patients their options during the care pathway and monitors them regularly, not only on their symptoms but also on their occupational issues and their and their relative’s need for information. This information must be calibrated to their actual condition and level of perceived self-efficacy that are, according to Bandura [23], developed and influenced through four primary sources of information: direct mastery experiences (performing an activity), vicarious experiences (observing others similar to oneself successfully performing an activity), social/verbal persuasion (being influenced to believe in ones’ capabilities to achieve a goal), and physiological state (emotional arousal when performing a new activity). That could be a pivotal coordination task for advanced OT practitioners that can facilitate the strategic use of resources and thus achieve a higher level of care [24].

The expressed wish of the former EME participants for a more profound thematization of cognitive fatigue and valuable strategies could be related to the fact that cognitive impairment is amongst the most common and debilitating symptoms of PCS (prevalence rate, 0.22; 95% CI, 0.17, 0.28) [25]. However, fatigue and cognitive impairment seem to be two distinct symptoms, with only 5% of patients suffering from both conditions [26]. To facilitate the OTs to lead the group interaction and the individual tasks appropriately, it would be helpful to obtain more information about possible limitations on a cognitive level of the EME participants. For that reason, it is essential to screen for cognitive impairment before starting EME and to offer additional individual OT to those with the presence of both symptoms. 

The EME therapists pointed out that handling the group and the expectations of its participants on the intervention is emotionally challenging, especially with people with PCS. Implementing a treatment protocol for a new patient population during an ongoing pandemic is a big challenge. The feelings of the OTs in this study align with other studies that reported positive and negative experiences of OTs in facing this new context [25]. They underlined the importance of an advance of trust on the part of the participants that the intervention will bear fruit. They are conscious that behavior changes take time and result from an individual’s intensive confrontation with his/her own doing. Providing general advice and quick solutions may appear effective but not increase self-management skills [26]. Sharing experiences with colleagues, patient information after discharge or results from clinical studies, and further delineating the concept of self-management [27] are essential elements to provide OTs with the confidence to be together with the participants on the right track. The training course for leading EME therapists should address these needs of empowerment in health promotion and education and provide evidence-based literature and instruments, e.g., a community of practice, quality circle, newsletter, and testimonials [28].

### Limitations

The potential biases within the study can significantly impact the reliability and generalizability of the findings:

Sample size and sampling bias: The limited number of EME participants due to scheduling and pandemic constraints, along with convenience sampling, raises concerns about the representativeness of the sample. The failure to achieve theoretical data saturation might indicate incomplete coverage of experiences, potentially leading to incomplete or skewed conclusions.

Data collection method and bias: The absence of more structured methodologies, visualization techniques, and varied question types may have contributed to unstructured and overarching comments, potentially leading to data that lack depth and precision. Additionally, the absence of representation from individuals with immigration backgrounds or languages other than German, as well as those with insufficient energy, introduces selection bias and limits the study’s applicability to diverse populations.

Online modality and interaction bias: The online format might have hindered the depth of interaction during discussions, impacting the quality and depth of insights shared during the focus groups.

Occupational therapists’ involvement: Many of them participated in an EME introduction course led by AW. There is a possibility that negative or ambiguous experiences with EME were underreported or downplayed due to participant motivation, leading to an incomplete picture of the intervention’s shortcomings or challenges.

Despite these limitations and biases, the study managed to gather valuable insights into the experiences of both patients and occupational therapists regarding a newly implemented intervention, providing a foundation for optimizing EME materials and guiding further training for occupational therapists. However, it is crucial to acknowledge and address these biases to ensure a more comprehensive and unbiased understanding of the intervention’s impact and efficacy.

## 5. Conclusions

The insights gathered from former participants of EME offer significant implications and potential applications for improving the support and management of individuals dealing with PCS.

The positive feedback highlighting the value of appreciation and respect within the peer group and from therapists during EME sessions underscores the importance of a supportive environment in aiding individuals’ understanding of fatigue and in implementing effective energy management strategies. The observed continuation of optimized daily routines post-EME, characterized by informed decision making in energy management, suggests that the program’s impact persisted beyond its completion.

Recommendations from participants, such as starting EME earlier, incorporating sessions for relatives, and focusing more specifically on PCS-related issues, provide actionable insights for program enhancement. Additionally, therapists’ suggestions to limit group sizes, refine workbooks for clarity, and simplify visual layouts align with optimizing program accessibility and effectiveness.

The value added by EME within an interdisciplinary rehabilitation setting suggests its potential in addressing critical aspects of managing daily routines for those with PCS. However, further research is imperative. Exploring the program’s effectiveness across various outcome dimensions will not only reinforce the confidence of participants and OTs in its efficacy but also validate the positive feedback received from individuals with PCS and therapists.

This feedback-driven approach, along with ongoing research to substantiate effectiveness across multiple dimensions, can bolster the refinement and broader implementation of programs like EME, potentially improving the quality of life and functional abilities for individuals navigating the complexities of PCS.

## Figures and Tables

**Table 1:** Structure and topics of the Energy Management Education (EME).

Structure	Delivery Modality/Duration	Topics
Session 1	Individual, face-to-face/45 min	Energy account
Session 2–6	Peer group (2–5 participants)/75 min plus	Break management
	15 min break/once per week	Occupational balance
		Use of body and environment
		Simplifying activities
		Effective communication
Session 7	Individual, face-to-face/45 min	My goals
Booster	e-mail/eight weeks after session 7	Review and reinforce

**Table 2:** Sociodemographic characteristics of participants with post-COVID syndrome.

Characteristics of EME Participants	
Gender, *n* (female/male)	6 (5/1)
Age, mean (SD)/min–max	54.7 (11.6)/41–73
Housing, *n*	
Cohabiting	5
With children	4
Level of education (school years), *n*	
Lower-secondary education (<12)	0
Upper-secondary education (12–16)	3
Tertiary level education (>16)	3
Current professional activity, *n*	
On medical leave	4
Full-time work	0
Part-time work	1
Unemployed	0
Retired	1
Family work	0
Months from COVID-19 infection to EME start, mean (SD)/min–max	11.2 (5.2)/6–20

*n* = number, SD = standard deviation, min = minimum, max = maximum.

**Table 3:** Results from focus groups with Energy Management Education participants.

Contexts	Themes	Categories
Intervention context: Interpersonal and dynamic interactions between person(s) and therapist and the shared occupational elements of therapeutic practices. Implementation content occurs in the intervention context, and the changes which occur as a consequence reshape the person-in-context [16] (p. 14).	Experiences with EME	Program Occupational Therapists Content of sessions Strategies and tools Self-training
	Recommendations for health professionals	Information Rehabilitation
Person-in-context expresses how context, person, and occupation arise together in mutually constitutive processes [16] (p. 14). Macro context: Includes many of the elements of the environment (e.g., government and political structures, technology, global events) that have an ongoing influence on person, therapist, and intervention contexts [16] (p. 14).	Living with long COVID	Long COVID
		After COVID-19 infection

## Data Availability

Data available upon request due to restrictions on privacy.

## Supplementary Materials

The following supporting information can be downloaded at https://www.mdpi.com/article/10.3390/healthcare12020150/s1, focus group discussion.
