# Supplementary material for: Energy Management Education in Persons with Long COVID-Related Fatigue: Insights from Focus Group Results on Occupational Therapy Approach
Source: Healthcare (Basel). 2024 Jan 9;12(2):150. doi: 10.3390/healthcare12020150 (PMC10815414; doi:10.3390/healthcare12020150)
Supplement: Supplementary file 1 [file healthcare-12-00150-s001.zip › healthcare-2777875-supplementary.pdf]

**Focus group discussion EME- participants post intervention.**

**Introductory question:** How did you experience the training on energy management?

**Key questions:**

A: Personal experience during the EME

a) How would you describe the energy management training to someone you know?
b) What was particularly interesting for you?
c) What did you struggle with? e.g., comprehensibility, applicability, instruction, group dynamics
d) How would you summarize your experience during the EME?

B: Appropriateness of topics and materials

a) How relevant was the content discussed to your current situation?
b) Are there aspects that you would have liked to deepen in your current situation?
c) How appropriate were the examples, worksheets and self-training exercises?
d) What could you apply and what was not feasible?

C: Suggestions for improvement

a) What do you think we should change about the EMS?
b) What suggestions for improvement do you have for ....?
   - Materials
   - Organization (duration, frequency)
   - content
   - Practical part during the lessons
   - Self-training exercises

**Final question:** Would you like to add anything else to what we have discussed today?

**Focus group discussion EME- participants 2-month post intervention.**

**Introductory question:** Retrospectively what would you like to say something about EME?

**Key questions:**

A: Energy management training in retrospect

a) Would you recommend EME to other people with long COVID-related fatigue? To whom? When? Why? Why not?
b) How do you deal with your energy today? Has anything changed?
c) If yes: what behavioral changes have you been able to implement?
d) Were there any topics in the overall training concept that were decisive for you in your conviction that you can change something? If yes: which ones?

B: Thoughts regarding effects on your everyday life & applicability

a) What aspects of the content have encouraged you to engage with the topic?
b) From your current perspective, what can you really use in your everyday life?
c) How practicable do you find the strategies and suggestions offered for your everyday life?

C: Suggestions for improving the therapy and/or the material

a) What should we pay more attention to?
b) Do you have any suggestions for improvement (in terms of content, structure, practical ...)? Which ones?

**Final question:** Is there anything else you would like to add to what we have discussed today?

**Specification questions**

- Can you give me an example of this?
- Can you explain .... in more detail? What exactly do you mean by ...?
- Tell me more about this!
- Why is this aspect important to you?

- Are there other/same opinions in the group on this aspect? Which ones?
- Do the other participants agree? Why/why not?

**Focus group discussion with EME leading OTs.**

**Introductory question:** How much experience have you had in treating people with long COVID-related fatigue so far?

**Key questions:**

A: Needs of people with long COVID-related fatigue

a) What is particularly important for people with long COVID-related fatigue?
b) What do you think ETs should pay particular attention to? In which lessons?
c) What has not changed for you?

B: Changes in the implementation

a) Have you had to adapt the content of the treatment protocol to the needs of this patient group? If yes: what do you do differently?
    - Access, assessments & lesson 1
    - Lesson 2-8
    - Inpatient setting: How do you solve L8? Do you do the reminder at all?
    - Outpatient setting: Do you offer L8? What have you adapted/changed?
    - Has the way you run the group changed? What do you do differently?
    - Does the time management work? If not, why?
    - Has the group dynamic between the participants changed? What should be considered?

C: Suggestions for improvement

a. What should be adapted or supplemented in terms of content?
b. What instructions and/or information for the ETs should be added/adapted in the manual?
c. What organizational/structural improvement suggestions do you have?

**Final question: Is there anything else you would like to add?**

**Specification questions**

- Can you give me an example?
- Can you explain .... in more detail? What exactly do you mean ...?
- Tell me more about it!
- Why is..
